# Firefighter Overexertion: A Continuing Problem Found in an Analysis of Non-Fatal Injury Among Career Firefighters

**DOI:** 10.3390/ijerph17217906

**Published:** 2020-10-28

**Authors:** Aurora B. Le, Lily A. McNulty, Mari-Amanda Dyal, David M. DeJoy, Todd D. Smith

**Affiliations:** 1 Department of Environmental Health Sciences, University of Michigan School of Public Health, Ann Arbor, MI 48109, USA; 2 Department of Applied Health Science, Indiana University School of Public Health–Bloomington, Bloomington, IN 47405, USA; lmcnulty16@gmail.com (L.A.M.); smithtod@indiana.edu (T.D.S.); 3 Department of Health Promotion & Physical Education, Kennesaw State University, Kennesaw, GA 30144, USA; mdyal2@kennesaw.edu; 4 Department of Health Promotion & Behavior, University of Georgia College of Public Health, Athens, GA 30602, USA; dmdejoy@uga.edu

**Keywords:** firefighter, occupational injury, overexertion, sprains and strains, musculoskeletal disorders

## Abstract

Traditionally, safety-related research on firefighting has focused on fires and fireground smoke as the primary source of non-fatal firefighter injury. However, recent research has found that overexertion and musculoskeletal disorders may be the primary source of firefighter injury. This study aimed to provide an update on injury occurrence among career firefighters. Injury data were collected over a two-year period from two large metropolitan fire departments in the U.S. Injury data were categorized based on the Bureau of Labor Statistics’ Occupational Injury and Illness Classification System. Cross-tabulations and Chi-square tests were used to determine the primary causes of injury, as well as the injury region. Between the two fire departments, there were 914 firefighters included in the analysis. The median age was 40.7 years old with those aged 40–49 as the largest age group for injury cases (38.3%). The most frequently reported cause of injury was ‘overexertion and bodily reaction’ (*n* = 494; 54.1%). The most reported injury region was in ‘multiple body parts’ (*n* = 331; 36.3%). To prevent subsequent musculoskeletal disorders that may arise due to overexertion, initiatives that promote enhanced fitness and ergonomics based on an analysis of the physical demands of firefighting are suggested.

## 1. Introduction

Firefighters (FFs) have always had a critical role in society as first responders who uphold public safety and health. As of 2018, the National Fire Protection Association (NFPA) estimated that there are approximately 1,216,000 firefighters in the United States, including career, volunteer, and paid-per-call FFs, as well as civilian staff and non-firefighting personnel [1]. In their roles, FFs not only respond to and suppress fires, but they also respond to medical emergencies and mitigate and manage adverse roadway and hazardous material events. Inherent to the dangerous nature of the profession, FFs are at an increased risk from a variety of physical and psychosocial workplace stressors, as well as increased potential for injury, illness, and fatality [2]. Moreover, with the increasing number of wildfires each year as a result of climate change spurring more land and structural fires, the physical and mental demands on FFs have intensified [3,4]. 

Traditionally, safety-related research on firefighting has focused on fires and fireground smoke as the primary source of non-fatal FF injury [5,6,7]. While the number of FF non-fatal injuries has notably decreased in the last few decades from over 100,000 in 1981 to 59,000 in 2017, injuries relating to musculoskeletal disorders have been on the rise and now comprise nearly half of the non-fatal injuries [8]. Overexertion is an exposure or event that can result in a non-impact injury or illness due to excessive physical effort; it is the way that the body responds or reacts to an external influence [9]. According to the Occupational Safety and Health Administration (OSHA), the term “overexertion” is sometimes used synonymously or interchangeably with “bodily reaction” [10]. Strains can be defined as injury to the muscle or musculotendinous joint, and sprains are an injury to the ligament [11]. Overexertion, which can cause these musculoskeletal disorders, is the leading cause of non-fatal injury across ten industry categories and accounts for 35% of non-fatal injuries and illnesses resulting in days away from work per year [9]. Moreover, it is estimated that overexertion and resultant sprain and strain injuries cost U.S. businesses and organizations an estimated USD 13.8 billion a year in direct costs, which include medical and lost-wage payments [12]. 

The firefighting industry is no exception to these costly and debilitating injuries and illnesses due to workplace exposures. Previous studies, which included part-time, volunteer, and other firefighting personnel, have aimed to characterize the extent of the impact of overexertion and strains and sprains on FFs. In a study of workers’ compensation claims (1992–1997) of FF municipalities in Illinois, over one-third of the reported injuries could be attributed to overexertion—typically of the lower back—and was subsequently identified as the primary source for FF workers’ compensation claims in those municipalities [13]. Another study utilizing data (2004–2009) from a fire department serving over 500,000 residents, also found that approximately one-third of injuries were due to exposures that resulted in strains and sprains; overexertion was identified as the leading cause of injury at this department [14]. Marsh and colleagues analyzed national non-fatal injury data (2003–2014) from the National Electronic Injury Surveillance System (NEISS–Work) occupational supplement, and found that overexertion and bodily reactions accounted for 20% of reported occupational injuries in U.S. FFs. [15]. It is thought that because career fire departments have more resources and programs, better equipment to address job demands, enhanced training, and firefighter fitness readiness, they might be in a position to better mitigate musculoskeletal injuries than volunteer or mixed-composition fire departments [1,16]. Several studies have attempted to characterize injury and fitness in career FFs. However, a uniform injury classification system that aligns with national data collection measures has not been utilized [17,18,19]. 

This study aimed to provide an update on injury occurrences specific to career firefighters, utilizing data from two large metropolitan U.S. cities’ fire departments implementing a uniform injury classification system to characterize reported non-fatal occupational injuries among firefighters.

## 2. Materials and Methods 

### 2.1. Data Sources and Setting 

Prior to the initiation of this research project, study protocols were approved by the Institutional Review Board at the University of Georgia (Study#: 2013104740). This was an injury epidemiological study aimed to determine the distribution of injuries and identify the determinants/causative factors of the injuries and safety related events in this specified population of career firefighters [20,21]. FF injury data and records were collected over a two-year period (May 2014 until May 2016) from two large metropolitan fire departments, one in the western U.S. (FD1) and one in the eastern U.S. (FD2), each serving populations greater than half a million people. The two departments were recruited for this study because of their similarities in operations, size, employee base, population served, etc. The two departments, situated in states on opposite sides of the U.S., were additionally selected to minimize potential bias that might occur if only one department in one geographic region was selected, and to account for wildland firefighting exposure. Western fire departments may more frequently encounter wildland fires because the wildand–urban interface is greater in the western U.S. Department comparisons are presented in Table 1. 

Each participating fire department provided copies of their FF injury reports. These reports did not identify the injured FFs. The data were entered into a dataset for analysis. Seven common variables, all of which were categorical, were available to researchers for comparison: year, date of injury, age, rank/occupation, station/shift, injury (specific), and cause. 

From the two fire departments, there were initially 69 unique classification statements for rank/occupation. Rank/occupation was collapsed into the following four categories based on commonly grouped ranks and chains of command within the fire service: ‘Firefighters’ (i.e., firefighters, recruits, engineers, firefighters/paramedics, sergeants); ‘Company officers’ (i.e., lieutenants, captains), ‘Chiefs’ (i.e., battalion, division), and ‘Other’ (i.e., accounting, grounds inspector, dispatcher) [1].

### 2.2. Classification of Injuries

Data on the specific body part(s)/region affected (neck, arm, etc.) initially had 187 unique classification statements from the two fire departments. The data were collapsed into categories based on the Bureau of Labor Statistics’ (BLS) Occupational Injury and Illness Classification System (OIICS) that provides a standardized coding system for characterizing work-related injuries. These 187 unique classification statements were collapsed into eight categories based on the BLS OIICS Trees v2.01 for Part of Body. The categories were: ‘Head’; ‘Neck, including throat’; ‘Trunk’; ‘Upper extremities’; ‘Lower extremities’; ‘Body systems’; ‘Multiple body parts’; and ‘Other body parts’ [22]. The ‘Other body parts’ classification included prosthetic and orthopedic devices. 

For the exposure or event that resulted in injury, the data initially had 773 unique classification statements from the two fire departments. These 773 unique codes were collapsed into eight categories based on the BLS OIICS Trees (company, city country) for Event or Exposure. The categories were: ‘Violence and other injuries by persons or animals’; ‘Transportation incidents’; ‘Fires and explosions’; ‘Falls, slips and trips’; ‘Exposure to harmful substances or environments’ (i.e., allergens, animals, plants); ‘Contact with objects and equipment’; ‘Overexertion and bodily reaction’; and ‘Other’ [22]. The unique classification statements were coded by the first author; a co-coder then reviewed all the coding designations and either agreed or disagreed. When there was disagreement between the two raters, consensus was achieved through further review and discussion. Initial disagreements occurred on approximately 1.6% (3/187) and 0.05% (4/773) of classifications/statements for specific body parts affected and cause of injury, respectively. 

### 2.3. Analysis 

The statistical analysis focused on the distribution of reported injury events based on age, rank, and specific body parts affected with cross-tabulations. Comparisons between groups were assessed using Chi-square tests. All data analysis was conducted using Stata 15.1 (StataCorp. 2017. *Stata Statistical Software: Release 15*, StataCorp LLC, College Station, TX, USA). 

## 3. Results

### 3.1. Demographics

**Figure 1:**
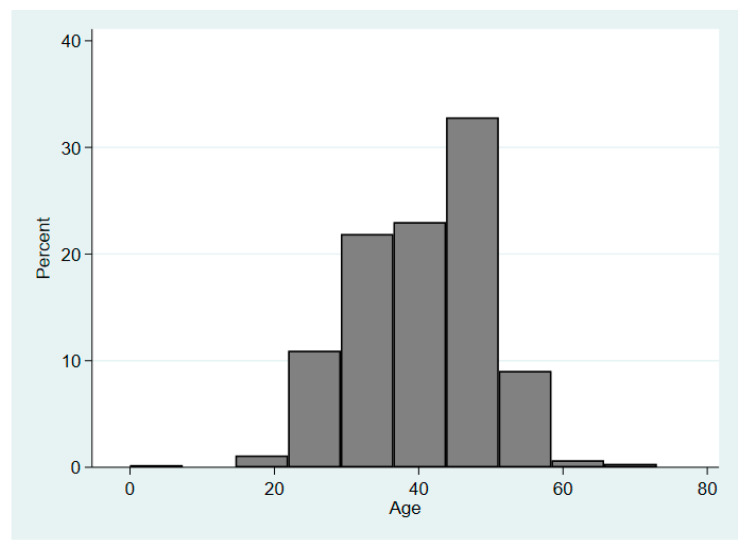
The combined age distribution of firefighters from fire department 1 (western U.S.) and fire department 2 (eastern U.S.).

**Figure 2:**
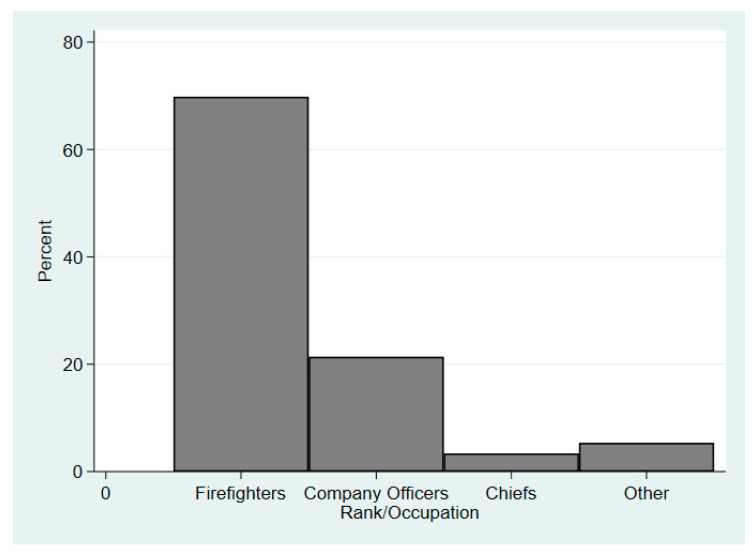
The combined rank/occupation distribution of firefighters from fire department 1 (western U.S.) and fire department 2 (eastern U.S.).

Of the 914 FF/personnel included in the analyses based on archival reported data/injury records, 59.6% (*n* = 545) were from FD1 and 40.4% (*n* = 369) were from FD2. The mean age across both departments was 40.73 years old (range 19–73) and the largest age group was 40–49 (*n* = 349; 38.3%) (Figure 1). The most predominant rank/occupation was firefighter (69.8%), followed by company officer (21.4%), chiefs (3.4%), and then other (5.4%) (Figure 2). 

### 3.2. Frequency, Causal Factors/Exposures of Injuries 

The frequency of casual factor/exposures across both fire departments are reported in Table 2 and the frequency of reported specific injury regions are reported in Table 3. Additionally, reported causal factors/exposures were cross-tabulated with age group, rank/occupation, and specific injury region. Relative to the total age distribution, FF personnel aged less than 39 experienced higher levels of injury as a result of fires and explosions, transportation incidents, and contact with objects and equipment, relative to those aged 40 and above. Those aged 40 and above experienced falls, slips and trips, as well as overexertion and bodily reactions at a higher prevalence than their younger counterparts. As the largest rank/occupation group, frontline firefighters had the highest prevalence of reported injury for each category of exposures/causal factors compared to the other ranks/occupations. There were no injuries reported for those aged 60 and above for fires and explosions, or transportation incidents. Across both fire departments, out of 914 personnel, only 24 were aged 60 and above, and the most common exposure/cause for injury among this age group was overexertion and bodily reaction (Table 4). 

Chi-square tests of independence showed there was a significant relationship between age and causal factor/exposures, *Χ*^2^ (35, *n* = 911) = 54.5, *p* = 0.019; there was a significant relationship between rank/occupation and causal factor/exposures, *Χ*^2^ (21, *n* = 911) = 73.3, *p* < 0.001; and there was a significant relationship between specific injury region and causal factor/exposures, *Χ*^2^ (49, *n* = 913) = 722.3, *p* < 0.001. Therefore, age, rank/occupation, and specific injury region had a relationship with the causal factor/exposures, respectively.

When comparing fire departments (FD1 and FD2), with the null hypothesis being that fire department and cause of injury are independent, there was a significant relationship between fire department and causal factors/exposures, *Χ*^2^ (7, *n* = 913) = 87.9, *p* < 0.001. Thus, we can conclude that there is a relationship between fire department and causal factors/exposures. Moreover, when comparing fire departments, with the null hypothesis being that fire department and specific injury region are independent, there was also a significant relationship between fire department and specific injury region, *Χ*^2^ (7, *n* = 913) = 470.2, *p* < 0.001. 

## 4. Discussion

This study aimed to provide an update to research conducted in the past decade which posits that overexertion, which can result in musculoskeletal disorders such as strains and sprains, is the primary source of non-fatal firefighter personnel injury and illness. Moreover, this study specifically focused on fire departments comprised of career firefighters. Given that career fire departments generally have more resources, enhanced training, and consistency in personnel relative to volunteer or mixed composition departments, they may be better suited to mitigate and manage musculoskeletal disorders [16,18]. Data aggregated from reported injury logs from two large metropolitan fire departments, each serving large populations, implementing a uniform injury classification system to characterize reported non-fatal occupational injuries among firefighters were used.

Between the two fire departments, there were 914 FF/personnel included in the analysis. These career FFs were predominantly middle-aged and identified as the rank of firefighter (including recruits, engineers, firefighters/paramedics) (Figure 1 and Figure 2). Based on the NFPA 2017 U.S. Fire Department profile, the largest age group for personnel was 30–39 (27.1%) followed by those aged 40–49 (23.7%), making the study sample slightly older than the national firefighter personnel age distribution [1].

When observing the distribution of causal factors/exposures resulting in a reported injury at both fire departments, overexertion and bodily reaction was the most frequent, followed by exposure to harmful substances or environments, and contact with objects and equipment (Table 2). When observing the distribution of the reported specific injury region, multiple body parts was the most frequent, followed by upper extremities and body systems (Table 3). This is congruent with recent literature and the NFPA’s 2017 firefighter profile [8,13,14,15]. With the median age of FFs being 40 in this study, our findings also corroborate results from multi-industry studies which show that musculoskeletal disorders as a result of overexertion are more prevalent among the middle-aged population. This may be due to lower levels of physical fitness than their younger colleagues, and to the physical degradation of joints, cartilage and connective tissues in the body that buffer against injury at younger ages. The physical degradation of the musculoskeletal system can result in more than one body part being affected [23,24,25]. Research has found that job demands, both physical and mental, are associated with the prevalence of musculoskeletal disorders in worker populations that are physically laborious, such as nurses and FFs. The higher the job demands and stress, the greater the likelihood of injuring oneself through overexertion. Furthermore, research demonstrates that psychosocial factors may influence injury rates among those in the fire service. Burnout can negatively influence safe work practices, resulting in more overexertion [2,23,26,27]. Additionally, it has been identified that a positive perception of safety climates can encourage safer behavior among FFs, and can lend to a greater sense of occupational calling, resulting in fewer injuries [28,29]. 

The cross-tabulation of causal factors/exposures of reported injury with the age, rank, and specific injury region (Table 4) demonstrated younger FFs were more prone to injury due to fires and explosions, transportation incidents, and injury due to contact and equipment compared to their middle-aged counterparts. As FFs became more aged and experienced, injuries were less of a frontline nature and more due to overexertion, bodily reaction, and falls, slips and trips. While firefighting personnel of all ranks are expected to maintain a certain level of physical fitness, injury due to overexertion resulting in strains and sprains may inevitably occur for a combination of reasons including but not limited to: injuries and bodily trauma potentially accumulating over time with the fire service; the physical effects of aging causing a loss of function and greater injury due to overexertion; becoming more sedentary as one completes candidate school; and higher rates of substandard fitness and obesity in career firefighters vs. volunteer firefighters [14,17,30]. To mitigate this, well-rounded wellness and health promotion programs in fire departments should be considered as they have the potential to decrease obesity and increase mental wellbeing—both contributing but preventable factors of musculoskeletal disorders [31]. Additionally, the NFPA 1582 Standard on Comprehensive Occupational Medical Programs for Fire Departments and NFPA 1583 Standard on Health-Related Fitness programs can be used by fire departments to improve their employees’ health and fitness by utilizing recommendations on aerobic capacity, body composition, muscular endurance, arm and leg strength, grip strength, flexibility, and other metrics to determine a standard of minimum physical fitness which career firefighters in their respective departments are expected to maintain [15,32]. It is recognized that the existence of these resources does not necessarily mean that the guidance is being implemented in career fire departments. Therefore, fire departments may also want to determine what physiological metrics are utilized for minimum job requirements. It would be appropriate for career fire departments to integrate the aforementioned guidance into their departmental expectations to not only reduce worker exposure to overexertion, but also potentially reduce the direct and indirect costs of treating musculoskeletal disorders.

The results from the chi-square tests of independence looking at the relationships between age, rank/occupation, and specific injury region with causal factor/exposures were significant. Moreover, when comparing FD1 and FD2, the chi-square tests showed that there was a relationship between the fire department, causal factors/exposures, and the specific injury region. Regardless of fire department, causal factors/exposures are inexorably tied to age, rank, and specific injury regions. To address the causal factors/exposures of FF injury, with overexertion being the prime cause, a multifaceted ergonomic intervention to mitigate and manage these exposures should be implemented. We suggest a combined initiative that promotes enhanced fitness and ergonomics based on a careful analysis of the physical demands of firefighting in tandem with the use of tools and equipment. One option is a participatory ergonomic program to increase worker awareness of ergonomics and/or evaluate solutions to optimize musculoskeletal loads in their work. Participatory ergonomics has been used in other physically demanding fields, such as commercial kitchens and carrier depots, and has proven to be effective in improving risk factors related to musculoskeletal disorders and meaningful worker participation [33,34,35,36]. A second option is to assess specific movement groups to granulize and target movement group to prevent injury among career FFs. Although novel research in ergonomic intervention design for specific tasks has begun focusing on fire services and emergency medical services, it has not explored in-depth specific tasks (e.g., maneuvering the fire hose, lifting individuals) related to movement groups and addressing those movement groups [14,37,38,39,40,41]. Ergonomic assessment of movement groups in other fields/occupations with highly variable tasks and high job-demands, such as nursing, military personnel, and police officers has proven to be successful in improving injury prediction and subsequently preventing it [38,42,43,44]. 

Further implications of these findings point to improvements that may be needed concerning equipment design, staffing, and teamwork models. By applying the Hierarchy of Controls within fire service practice and determining how equipment selection and design influences bodily reactions, it can be determined if personal protective equipment selection can be modified to decrease strain and enhance physical performance, whether station design can be modified, if more frequent shift rotation is required, and if more team-assisted tasks are needed [45,46,47]. Targeting ergonomics through exercise, education, and treatment may be more feasible to initially implement. While it may be challenging and costly to change engineering controls for fire departments that have multiple stations, it could provide a return on the investment in the long-term [11].

Lastly, firefighting associations and professional organizations (e.g., National Fire Protection Association, International Association of Fire Chiefs) could more strongly advocate for improved, standardized data collection of non-fatal FF injury and illnesses, as well as fatalities, so that reporting of these incidents is not only enhanced but the data can be better analyzed to compare across states and departments, as well as controlling for potential confounding factors. A data collection system that could be used as a model is the National Fire Incident Reporting System (NFIRS), established by the National Fire Data Center of the U.S. Fire Administration to provide “uniform coding for fire protection” [48]. Although participation in the NFIRS is voluntary, it still manages to capture approximately 75% of all reported fires that occur annually through its 23,000 participating fire departments. Collecting robust, uniform data on FF injury could provide departments with data to use for better identifying which health and safety issue should be addressed and permits continuous improvement, just as this study attempted to do by utilizing the BLS OIICS as a guide for a standardized coding system for characterizing work-related injuries

There are limitations to this study that need to be acknowledged. Firstly, the data provided from both fire departments did not provide any information on gender, race/ethnicity, or any other potential confounding factors (e.g., fitness level, physical and mental comorbidities, amount of training received) given that the researchers were provided de-identified data. Secondly, the data from the fire departments lacked details about the nature of the injuries; a standardization of metrics or descriptor categories (i.e., similar to that available through the National Fire Incident Reporting System) could be adapted. Thirdly, there was no standardization of metrics or descriptor categories, so all the variables in this dataset were categorical. Lastly, data were collected from two large metropolitan fire departments and therefore may not be generalized to smaller fire departments.

## 5. Conclusions

With the increasing numbers of outdoor and indoor fires in the U.S., the physical and psychological demands on firefighters is unlikely to decrease in the coming decade. With these increased demands, increased injury rates are likely to follow suit. This update on non-fatal injury rates among career firefighters using data from two large metropolitan fire departments confirms that it is not fires and burns that are the greatest source of injury for firefighting personnel, but rather overexertion. Greater consideration should be taken for combined initiatives that promote ergonomics, conduct ergonomic assessment on movement groups, encourage enhanced fitness standards, and develop analysis of firefighters’ physical demands in tandem with the use of their equipment to identify issues and ultimately decrease the costly but preventable musculoskeletal disorders that arise as a result of overexertion.

## Figures and Tables

**Table 1:** Comparison of participating fire departments.

Department Characteristics	FD1 (Western U.S.)	FD2 (Eastern U.S.)
Number of Stations	33	35
Number of Personnel	~750 sworn firefighters	~1000 sworn firefighters
Number of Calls per year	~95,000	~100,000
Population Served	~1.2 million	~519,000
Square Miles Protected	181	132
International Airport Protected?	Yes	Yes

**Table 2:** Reported causal factor/exposure resulting in injury.

Causal Factor/Exposure	Count	Percentage
**Overexertion and bodily reaction**	494	54.1
**Exposure to harmful substances or environments**	150	16.4
**Contact with objects and equipment**	103	11.3
**Falls, slips, and trips**	79	8.7
**Violence and other injuries by persons or animals**	24	2.6
**Transportation incidents**	23	2.5
**Fires and explosions**	18	2.0
**Other**	22	2.4

**Table 3:** Reported specific injury region.

Injury Region	Count	Percentage
**Multiple body parts**	331	36.3
**Upper extremities**	173	19.0
**Body systems (i.e., circulatory, nervous, immune)**	121	13.2
**Trunk**	120	13.1
**Lower extremities**	103	11.3
**Head**	24	2.6
**Neck, including throat**	12	1.3
**Other**	29	3.2

**Table 4:** Distribution of causal factors/exposures of reported injury by age, rank, injury region, 2014–2016.

	Causal Factor/Exposure, *n* (%)								
	Contact with Objects and Equipment	Exposure to Harmful Substances or Environments	Falls, Slips, and Trips	Fires and Explosions	Overexertion and Bodily Reaction	Transportation Incidents	Violence and Other Injuries by Persons or Animals	Other	Total
Age Group									
18–29	17/103 (16.5%)	26/150 (17.3%)	5/78 (6.4%)	4/18 (22.2%)	44/494 (8.9%)	5/23 (21.7%)	5/23 (21.7%)	2/22 (9.1%)	108/911 (11.9%)
30–39	33/103 (32.1%)	63/150 (42.0%)	19/78 (24.4%)	7/18 (38.9%)	146/494 (29.6%)	8/23 (34.9%)	7/23 (30.5%)	3/22 (13.6%)	286/911 (31.4%)
40–49	31/103 (30.1%)	48/150 (32.0%)	37/78 (47.4%)	4/18 (22.2%)	205/494 (41.5%)	5/23 (21.7%)	6/23 (26.1%)	13/22 (59.1%)	349/911 (38.3%)
50–59	19/103 (18.4%)	12/150 (8.0%)	16/78 (20.5%)	3/18(16.7%)	82/494 (16.6%)	5/23 (21.7%)	4/23 (17.4%)	3/22 (13.6%)	144/911 (15.8%)
60–64	1/103 (1.0%)	0	0	0	3/494 (0.6%)	0	0	0	4/911 (0.4%)
65 and above	2/103 (1.9%)	1/150 (0.7%)	1/78 (1.3%)	0	14/494 (2.8%)	0	1/23 (4.3%)	1/22 (4.6%)	20/911 (2.2%)
Rank/Occupation									
Firefighters	68/103 (66.0%)	119/150 (79.3%)	52/79 (65.8%)	15/18 (83.3%)	349/493 (70.8%)	12/23 (52.3%)	11/23 (47.8%)	10/22 (45.5%)	636/911 (69.8%)
Company officers	23/103 (22.3%)	28/150 (18.7%)	17/79 (21.5%)	2/18 (11.1%)	108/493 (21.9%)	5/23 (21.7%)	8/23 (34.8%)	4/22 (18.2%)	195/911 (21.4%)
Chiefs	4/103 (3.9%)	1/150 (0.7%)	2/79 (2.5%)	0	20/493 (4.1%)	3/23 (13.0%)	0	1/22 (4.5%)	31/911 (3.4%)
Other	8/103 (7.8%)	2/150 (1.3%)	8/79 (10.2%)	1/18 (5.6%)	16/493 (3.2%)	3/23 (13.0%)	4/23 (17.4%)	7/22 (31.8%)	49/911(5.4%)
Injury Region									
Head	9/103 (8.8%)	4/150 (2.7%)	0	5/18 (27.7%)	3/494 (0.6%)	1/23 (4.8%)	0	2/22 (9.1%)	24/913 (2.6%)
Neck	2/103 (1.9%)	0	0	1/18 (5.6%)	4/494 (0.8%)	5/23 (23.8%)	0	0	12/913 (1.3%)
Trunk	6/103 (5.8%)	0	4/79 (5.1%)	1/18 (5.6%)	105/494 (21.3%)	2/23 (9.5%)	1/24 (4.2%)	1/22 (4.5%)	120/913 (13.2%)
Upper extremities	23/103 (22.3%)	2/150 (1.3%)	3/79 (3.8%)	0	142/494 (28.7%)	1/23 (4.8%)	2/24 (8.3%)	0	173/913 (18.9%)
Lower extremities	10/103 (9.7%)	1/150 (0.7%)	15/79 (18.9%)	0	71/494 (14.4%)	5/23(14.3%)	1/24 (4.2%)	0	103/913 (11.3%)
Body systems	3/103 (2.9%)	82/150 (54.7%)	0	2/18 (11.1%)	13/494 (2.6%)	1/23 (4.8%)	17/24 (70.8%)	3/22 (13.6%)	121/913 (13.2%)
Multiple parts	50/103 (48.6%)	44/150 (29.3%)	56/79 (70.9%)	9/18 (50.0%)	147/494 (29.8%)	6/23 (28.5%)	3/24 (12.5%)	16/22 (72.8%)	331/913 (36.3%)
Other	0	17/150 (11.3%)	1/79 (1.3%)	0	9/494 (1.8%)	2/23 (9.5%)	0	0	29/913 (3.2%)
